# Supporting Individuals With Cognitive Impairment and Family Members in Rural Communities: Protocol for a Mixed Methods Digital Health Study

**DOI:** 10.2196/77958

**Published:** 2025-10-06

**Authors:** Eric S Cerino, Megan C McCoy, Margarita Martinez, Thomasina J Seaton, Rasheera Dopson, Travis J Anderson, Gillian Porter, Faith R Shannon, Raechel A Livingston, Amanda D Black, Jacqueline Mogle, Louis Lucero, Michael J McCarthy

**Affiliations:** 1 Department of Psychological Sciences, College of Social and Behavioral Sciences, Northern Arizona University, 1100 S. Beaver Street, Flagstaff, AZ, 86011, United States, 1 9285232662; 2 Interdisciplinary Health Program, College of Social and Behavioral Sciences, Northern Arizona University, Flagstaff, AZ, United States; 3 Department of Social Work, College of Social and Behavioral Sciences, Northern Arizona University, Flagstaff, AZ, United States; 4 RTI Health Solutions, Research Triangle Park, NC, United States; 5 Joe C. Montoya Community and Senior Center, Flagstaff, AZ, United States

**Keywords:** mixed methods, digital health, rural health, community-engaged research, community-based participatory research, cognitive impairment, caregiving, dementia, mild cognitive impairment, subjective cognitive decline

## Abstract

**Background:**

The health and economic burdens of Alzheimer disease and related dementias (ADRD) are exacerbated for people living in rural social contexts who experience geographic barriers to care. There are currently few resources specifically designed to support socioculturally diverse rural ADRD care dyads, including early detection of potential precursors to ADRD such as mild cognitive impairment (MCI) and subjective cognitive decline (SCD).

**Objective:**

The primary objective of the Northern Arizona Memory Study (NAZMS) is to develop culturally informed and scalable resources to identify and support rural families at risk for ADRD. The purpose of this study is to introduce the NAZMS protocol and discuss its role in addressing dementia risk and promoting cognitive health in rural communities.

**Methods:**

This dyadic study will use a mixed methods, digital health approach. A sample of rural care dyads with MCI or SCD will be screened and recruited through partnerships with community centers across Northern Arizona. Consenting dyads will complete semistructured interviews to answer questions about technology preferences for monitoring symptoms and engaging in remotely delivered interventions. Next, care dyads will complete separate baseline questionnaires assessing dyadic (eg, experiences with caregiving or care receiving) and health factors. Participants with cognitive impairment will then complete a 14-day mobile protocol of brief end-of-day surveys and cognitive assessments delivered via study-provided smartphones.

**Results:**

Data from the qualitative interviews will provide dyad preferences for intervention development. Data from the quantitative protocol will specify for whom (ie, between-person) and on which days (ie, within-person) modifiable factors are related to better cognitive health in everyday life.

**Conclusions:**

This study will take a mixed methods, digital health approach to supporting rural families at risk for ADRD by understanding intervention preferences and identifying the modifiable protective and risk factors that influence cognitive health in everyday life. The findings are expected to directly support rural Arizonans and respond to national priorities in ADRD research for the development of community-based disease education programs and the use of digital assessments of cognitive health and well-being.

## Introduction

### Background

The aging of the US population has brought an unprecedented increase in the prevalence of Alzheimer disease and related dementias (ADRD). More than 6 million individuals in the United States live with ADRD, and this number is expected to increase dramatically in the coming years [1-3]. ADRD is costly, with annual direct medical expenditures calculated to be US $14,508 per older adult with ADRD versus US $10,096 per older adult without ADRD [2]. Individuals with ADRD may exhibit disruptive cognitive and behavioral symptoms such as impaired memory and decision-making, confusion, and agitation several years before the disease is recognized and supports are put into place [4]. This is distressing for the individual facing these symptoms, as well as untrained caregivers who often serve as the primary source of daily support. Caregivers are at high risk for health problems such as depression, poor sleep quality, high blood pressure, and heart disease [5-8]. This situation is exacerbated for care dyads (ie, people with cognitive impairment and their care partners) living in rural social contexts who are isolated [9], have few resources [10], experience geographic barriers to care [11], and lack basic access to ADRD-related information and services [12,13]. Compared with urban areas, rural individuals with ADRD experience more days in nursing homes and significantly higher mortality [14,15]. The likelihood that cases of ADRD in the United States are underdiagnosed and undercounted complicates our understanding of this emerging public health problem and leads to additional challenges for families [9,16].

Lack of access to specialty care (ie, neuropsychological evaluations) and geographic or transportation barriers make timely detection and early screening of cognitive impairment an ongoing challenge [17,18]. Local community centers in Northern Arizona offer unique potential to compensate for the lack of formal services in memory care deserts [17] and rural areas [18], but need to be more intentionally integrated into community-based screening efforts to address this gap. Community-based participatory approaches in rural communities can foster relevant service delivery (eg, screening, referrals, and brain health education), strengthen care networks, and support sustainability [19]. Local community centers for older adults are ideally situated to support brain health in socioculturally diverse rural communities because they serve as trusted resource hubs for older adults and caregivers [20]. Further, older adults from historically minoritized communities may be distrustful of formal health care systems or researchers and place more trust in community-based organizations [21,22], reinforcing the value of these networks.

There are currently few resources and interventions specifically designed to identify and support socioculturally diverse rural ADRD care dyads, including protocols for the early detection of potential precursors to ADRD, such as mild cognitive impairment (MCI) or subjective cognitive decline (SCD), and validated strategies for community-based delivery of support [23,24]. Early detection of MCI and SCD is crucial for maximizing the effectiveness of interventions and clinical trials while efficiently allocating resources toward people at greater risk of transitioning to Alzheimer disease (AD) [25]. Without early detection and intervention, diverse families living in rural areas are left to cope in isolation, leading to devastating individual, interpersonal, and community-level consequences. Further, decreasing risk factors and increasing protective factors for ADRD, MCI, and SCD can prevent or delay up to 40% of ADRD cases [26].

The Northern Arizona Memory Study (NAZMS) applies a preventive digital health approach to develop technologies and resources necessary to identify and support rural dyads with MCI or SCD at risk for ADRD. Our work is situated within the cognitive decline and impairment stage of the typical disease progression of AD, beginning with preclinical AD (ie, no observable symptoms but potential for changes in the brain), followed by SCD (ie, self-reported difficulty and decline in memory and thinking domains) and MCI (ie, mild impairment in objective performance across multiple domains), and ending in mild, moderate, and severe stages of Dementia due to AD [1]. Specifically, NAZMS focuses on individuals with MCI or SCD and their caregivers because these individuals are at an elevated risk of transitioning to ADRD. Early detection of SCD and MCI is crucial for maximizing the effectiveness of interventions and clinical trials while efficiently allocating resources toward people at greater risk of transitioning to AD [27]. Addressing SCD is especially relevant for diverse communities in Northern Arizona, as one in six Indigenous adults reports SCD, with nearly two-thirds of these individuals reporting their SCD interferes with day-to-day activities [27]. Among the 22 federally recognized tribes in Arizona, many Tribal Nations have lands and strong cultural connections to Northern Arizona, including the Hopi Tribe, Navajo Nation, Hualapai Tribe, Havasupai Tribe, Kaibab-Paiute Tribe, and San Juan Southern Paiute Tribe [28]. Our community-based participatory approach to identifying and supporting care dyads emphasizes community strengths and self-determination, as well as care dyads’ ability to express agency in their health care and in life in general.

By directly collaborating with local community centers for our study’s recruitment and outreach, we align our study to the unique needs, cultural norms, and practical realities that will ensure culturally informed supports and resources that are relevant for communities across Northern Arizona [29]. Our digital health study design and focus for future intervention development were further informed by the technology acceptance model for older adults, which recognizes that social context, user intention, perceived and confirmed usefulness, and ease of learning and use impact older adults’ acceptance or rejection of technologies [30]. The dyadic nature of NAZMS was informed by the developmental-contextual model of coping [31], which posits that dyadic illness appraisal (eg, illness ownership—yours or mine vs ours) and dyadic coping (eg, problem- and emotion-focused support) are key drivers of adjustment for care dyads. In this way, NAZMS ensures that both care partner and care receiver share their lived experiences, preferences, and insights throughout the study protocol.

### Objective and Aims of NAZMS

The primary objective of NAZMS is to develop culturally informed and scalable resources to identify and support rural families at risk for ADRD. The purpose of this study is to introduce the NAZMS protocol and discuss its role in addressing dementia risk and promoting cognitive health in rural communities. NAZMS uses a dyadic mixed methods, digital health approach to address the following two aims: (1) determine culturally appropriate care dyad preferences for identifying and monitoring cognitive and behavioral symptoms of MCI and SCD, and for engaging in remotely delivered supportive interventions to contend with or reduce symptoms and associated problems from MCI and SCD; and (2) examine the impact of modifiable multilevel (eg, individual, dyadic, family, and environmental) risk and protective factors on daily cognitive health among rural individuals with MCI or SCD.

## Methods

### Overview

NAZMS uses a convergent mixed methods design [32,33], in which qualitative and quantitative data are collected separately and then merged to develop a comprehensive understanding of rural care dyads at risk of ADRD.

### Participants and General Procedure

We are recruiting an initial target of 30 rural care dyads with MCI or SCD, who are interviewed [33,34] and purposively recruited [35] through partnerships with community centers in the 4-county Northern Arizona region. Recruitment efforts include local newspaper and community center advertisements (eg, flyers) and announcements made at community centers by center staff and study team members. The physical addresses of the community centers and participant homes were entered into the Health Resources and Services Administration rural eligibility tool [36] to determine rurality.

Target individuals (“people with memory or thinking challenges”) are included if they (1) live in the community, not in a nursing home; (2) score below 19 on the Montreal Cognitive Assessment (MoCA) Blind or report presence of decline in memory or thinking or concern in these domains; and (3) can identify a primary caregiver who is able and willing to participate in the study (spouse, adult child, other family member or friend, or family of choice). Both dyad members must be (1) capable of using technology with coaching and support; and (2) able to communicate in English and provide informed consent. Dyads are excluded if either dyad member is (1) under the age of 18 years; (2) pregnant; (3) has untreated severe mental illness, alcohol or drug abuse, or suicidality; or (4) has another neurological condition that significantly impacts function. Participants were considered capable of using technology with coaching and support if they indicated their willingness and capacity to engage with study-provided technology (eg, tablet for baseline survey and smartphone for 14-day daily diary) alongside trained study team members (eg, coaching and support). Exclusion criteria were applied to reduce the risk of confounding variables and health statuses that may obscure the focus of cognitive functioning in SCD or MCI due to AD [37,38]. If both dyad members experience MCI or SCD, the individual with the lower MoCA Blind score or more SCD is considered the target individual for this study.

**Figure 1.**
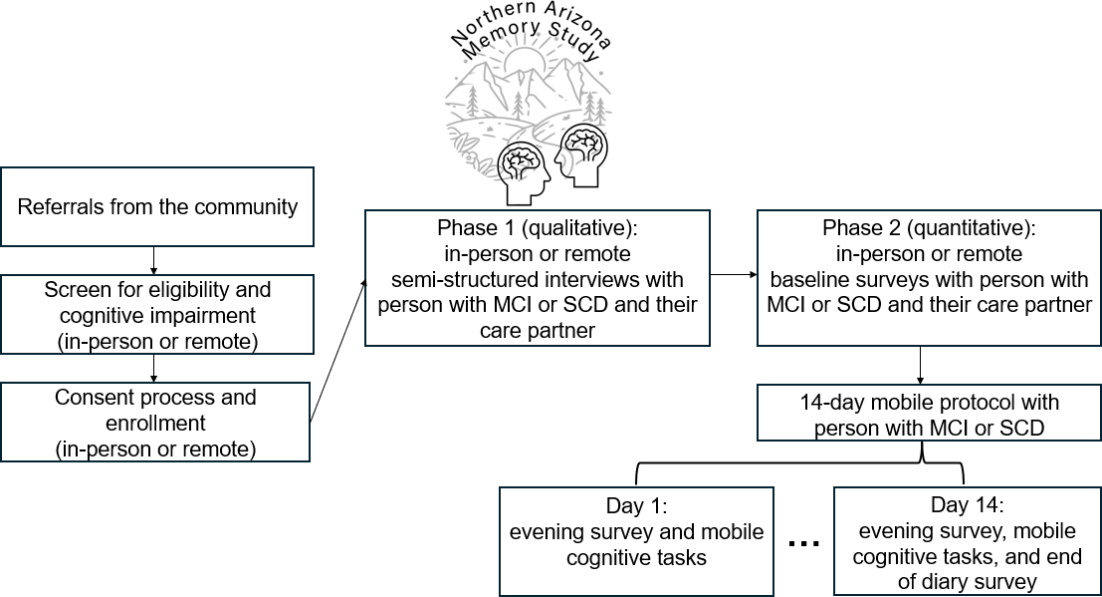
Visual timeline of the dyadic Northern Arizona Memory Study (NAZMS) protocol using a mixed methods, digital health approach. Following community referral, individuals are screened for eligibility and complete the consent process. Phase 1 data collection includes qualitative in-person or remote semistructured interviews with the person with mild cognitive impairment or subjective cognitive decline and the care partner. Phase 2 includes quantitative in-person or remote baseline surveys with both dyad members. The person with mild cognitive impairment or subjective cognitive decline is then provided with a study smartphone to complete a 14-day mobile protocol. MCI: mild cognitive impairment; SCD: subjective cognitive decline.

Upon completion of the semistructured interview, care partners and individuals with cognitive impairment complete a baseline questionnaire. The participants with cognitive impairment are then invited to participate in the quantitative 14-day daily diary component of the study. Recruitment and data collection began in fall 2024 and will continue throughout 2026. Figure 1 provides a visual timeline of the NAZMS protocol from community referral to in-person or remote participation in the mixed methods protocol.

The technology support provided by trained study team members and the mixed methods, multicomponent nature of our protocol support participation in NAZMS across a wide spectrum of technology proficiencies and cognitive statuses. We provide the cognitive screening as a free community service without a requirement to enroll in the NAZMS protocol. This screening does not require tablet or smartphone technology use, as it can be conducted in person with responses provided to a team member. The qualitative arm of the protocol does not require any technology use from the respondents who participate in person, and care partners can assist with technology connections for qualitative interviews conducted on Zoom (Zoom Video Communications, Inc). As long as the participant is open to receiving coaching and support from study team members (eg, hands-on training and troubleshooting phone calls and emails), they can participate in the quantitative arm of the protocol. NAZMS is a first step in developing supports and resources for rural care dyads at risk for dementia. This study can lay the foundation for future work that expands modalities of protocol administration (eg, mailed paper-and-pencil booklets and more frequent road trips to rural communities for in-person administration) and language accessibility (eg, Spanish, Native languages within nearby Tribal Nations) to better represent populations across Northern Arizona.

### Materials and Specific Procedures

#### Screening Protocol

Community-dwelling adults with cognitive impairment are screened using the MoCA Blind [39] to screen for MCI and adapted items from the Einstein Aging Study Health Self-Assessment [40,41] to screen for SCD. Participants are considered potentially eligible with MCI if they score ≤18 out of 22. The MoCA Blind was selected because it can be administered both in-person and via telephone, and it accommodates participants with vision or motor limitations. In contrast to the full MoCA scoring subcategories of mild, moderate, and severe impairment, the MoCA Blind has only been validated to distinguish cognitively unimpaired from potential MCI (scores ≤18/22). Thus, it is not possible to classify severe impairments consistent with dementias using this version of the MoCA. Participants unable to provide informed consent for the qualitative or quantitative arms of data collection will not be permitted to participate (this will likely refer to individuals with dementia who receive our screening). Participants answer items on perceived decline in memory and concern about these changes in memory (4 questions), perceived decline in thinking abilities and concern about these changes in thinking (4 questions), and 1 question on whether they have discussed their memory or thinking challenges with a health care provider. Participants are eligible with SCD if they report a decline in memory or thinking and/or concern in these domains.

#### Qualitative Protocol–Semistructured Interview

Textbox 1 provides the semistructured interview guide for the NAZMS qualitative protocol. Team members participated in training and practice sessions with the co-principal investigators to ensure familiarity with the semistructured interview process and engagement with people with MCI or SCD and their care partners. The semistructured nature of the interview allows flexibility to accommodate cognitively unimpaired participants and those with impairment (eg, interviewers had the questions as a road map, but did not need to ask every question in the same way to each participant). Questions were divided into a section on technology (eg, “What are your thoughts about if and how these kinds of technologies could be used to monitor memory or thinking challenges?”) and a section dedicated to memory and thinking challenges (eg, “What things have helped you to access support for memory or thinking challenges?”). Interview questions about dyad preferences for identifying and monitoring symptoms, as well as engaging in remotely delivered interventions, build on previous work with care dyads for neurological conditions (eg, stroke survivor–family caregiver dyads) [42,43] and are informed by the technology acceptance model for older adults [30] and the developmental-contextual model of coping [31].

Textbox 1. **Semistructured qualitative interview guide.**

**Prompts on technology:**

1. What kinds of technology do you use in your everyday life? Some examples include: landline phone, cell phone, iPhone or other Android smartphone, tablet computer, email, home computer, internet websites, social media (Instagram, Snapchat, and Facebook), and Zoom or FaceTime video calls.
2. How easy are these technologies for you to use?
3. How confident are you using these technologies?
4. What would make you feel more confident using these technologies?
5. What are some advantages you see to each of these technologies when it comes to communicating with family, friends, health care providers, etc?
6. What are some disadvantages?
7. What are your thoughts about whether and how these kinds of technologies could be used to monitor memory or thinking challenges for yourself or the person participating in the study with you?
8. What advantages and disadvantages of these technologies can you imagine for monitoring memory or thinking challenges?
9. What do you think about technologies for delivering support for memory or thinking challenges remotely (not face-to-face)? That is, to provide support, education, and tools for remembering things?
10. What advantages of these technologies can you imagine for providing support?
11. What about disadvantages?
12. What might help you to use these technologies to get support for memory or thinking challenges?
13. What might hinder you from using these technologies to get support for memory or thinking challenges?

**Prompts on memory or thinking challenges:**

Now we would like to talk about the memory or thinking challenges that you or your family member faces. Some examples of memory or thinking challenges include forgetting to do things, trouble staying focused or concentrating, misplacing items in your home, finishing everyday tasks such as paying a bill, losing your train of thought, having trouble following a conversation, finding it hard to make decisions, finishing a task or following instructions, or having poor judgment.

1. What are some symptoms of these memory or thinking challenges that you’ve noticed either in yourself or in the person participating in the study with you?
2. Tell me about what it has been like for you to deal with this.
3. In what ways have memory or thinking challenges impacted your everyday life? Practically speaking, as well as any emotional impacts they may have.
4. How have they impacted the person participating in the study with you, including your relationship with that person?
5. How do you communicate with the person participating in the study with you about memory or thinking challenges?
6. What strategies do you use to deal with memory or thinking challenges?
7. What have your experiences been accessing support to deal with memory or thinking challenges? For example, from health care or social service providers, friends, or other family members, etc.
8. What things have helped you to access support for memory or thinking challenges?
9. What things have hindered you from accessing support for memory or thinking challenges?
10. What advice do you have for other people with memory or thinking challenges and their family members as they deal with symptoms and seek outside support?

#### Quantitative Protocol–Baseline Survey

Upon completion of the semistructured interview, caregivers and individuals with cognitive impairment complete their own baseline questionnaires assessing *s*ociodemographic and health factors, caregiving, and technology preferences (Table 1). If participants have vision or motor limitations, a trained study team member can read the survey (ie, questions and answer options) directly to the participant and score responses. We assess a wide range of sociodemographic and health factors (eg, age, race, distance and transportation used for medical appointments, and mental health) to understand individual differences in preferences for supports, resources, and factors associated with daily cognitive health. Caregiver characteristics (eg, age, gender, and relationship), demands (eg, symptoms, types of care needed, hours spent providing care each day, and strain), cultural justification for providing care, and dyadic coping styles are assessed to evaluate the dyadic composition of caregiver and care receiver. We also obtain ratings of preferred symptom identification and monitoring technologies, intervention technologies, and data use considerations and comfort using an investigator-designed form, similar to what we have done in previous studies [42]. Table 1 provides additional details on the quantitative measures assessed at baseline.

**Table 1.** Baseline survey measures.

Target and construct	Response options	Items and operational detail	Relevant reference
Sociodemographic and health factors			
Sociodemographic factors	Checkbox or radio button	Age, ethnicity, race, gender, sexual orientation, birthplace, education, employment status, marital status, languages spoken at home, adults and children in home, health insurance status, number of medical visits in the last year, distance and transportation used for medical appointments, and household income	13
Health	Multiple options	General health (excellent-poor) and physical and mental health (days with poor health)	44
Anxiety	Not at all–nearly every day	Generalized Anxiety Disorder (GAD-2)	45
Depression	Not at all–nearly every day	Patient Health Questionnaire (PHQ-2)	45
Perceived control	Strongly disagree–strongly agree	1-item on global perceived control	46
Cultural practices	Multiple options	Religiosity, spirituality, prayer frequency, and the importance of cultural heritage	47 48
Caregiving			
Characteristics	Multiple options	Relationship type, diagnosis, length of time providing the care, and distance from care receiver	13
Demands	Multiple options	Symptoms, types of care needed, hours spent providing care each day, and burden	49 50
Informal supports	Checkbox	Types of informal services received from unpaid friends or family	47
Formal supports	Checkbox	Types of formal services received from paid professionals	47
Cultural beliefs	Strongly disagree–strongly agree	10-item Cultural Justification for Caregiving Scale	51
Dyadic coping	Very rarely–very often	10items from the Dyadic Coping Inventory	52
Technology preferences			
Access	Checkbox	Which technologies do you have access to?	43
Usability	Checkbox	Which technologies do you know how to use?	43
Data use considerations	Checkbox	Which types of data would you consider allowing researchers to use to answer research questions about memory and aging? (activity monitoring, heart rate, bed-based sleep sensor, and geolocation [GPS])	53
Data use comfortability	Extremely uncomfortable–extremely comfortable	Comfort with each type of data being used to answer research questions about memory and aging	53

#### Quantitative Protocol–14-Day Diary Design With Mobile Cognitive Tests

The participants with cognitive impairment are then invited to participate in the quantitative daily diary component of the study. All participants who agree to participate in the mobile protocol receive instructions and a mobile device preloaded with the quantitative survey and cognitive assessment application. If participants complete the interview and baseline survey in person at a community center, the kit of study supplies is provided to them in person. If the participant completes the protocol remotely on Zoom, the kit of study supplies is mailed to the participant. A 30-minute training is provided with the option of in-person or remote (ie, Zoom) administration to facilitate familiarity with the protocol and provide an opportunity to answer any questions before beginning the protocol. The collection kit includes an instructional sheet on how to complete the mobile protocol and contact information to use if they encounter any problems with the mobile device. A trained study team member is available to answer any technology-based questions from dyads, assist with setup, and provide any troubleshooting that may emerge during the mobile protocol.

**Figure 2.**
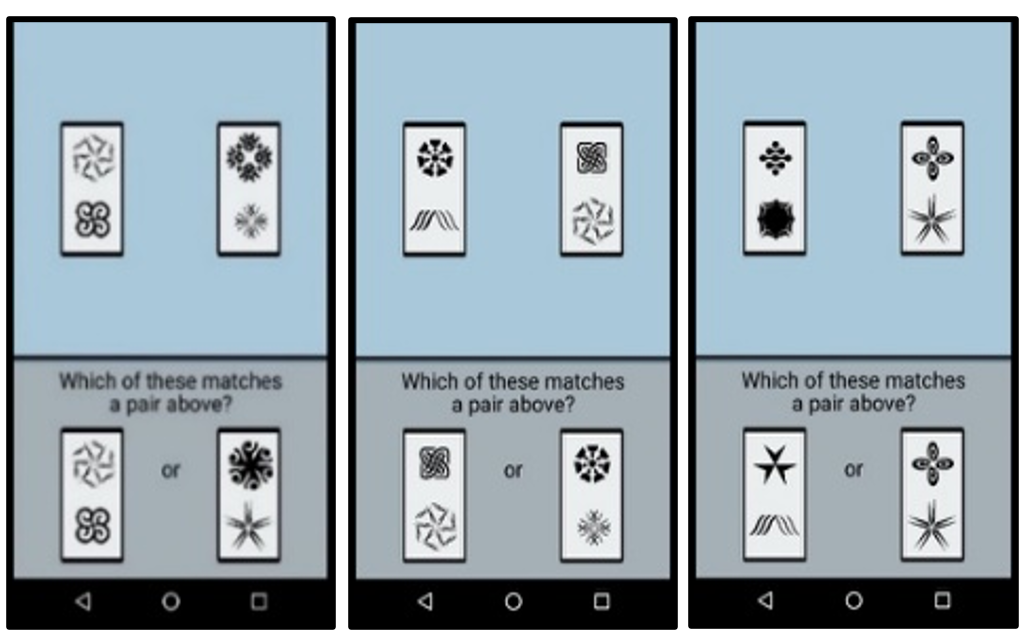
Screenshots of symbol match mobile cognitive task. Participants complete this cognitive task during the evening assessment for 14 consecutive days.

The nightly mobile survey asks about the participant’s daily experiences, with questions about contextual factors, daily stress, daily events, time use, daily psychosocial experiences, daily physical health indices, and daily cognitive health. The evening survey measures were adapted from the National Study of Daily Experiences (eg, [54]) to facilitate cross-study harmonization between this rural sample of care dyads at risk for ADRD and a national adult lifespan sample with up to 30 years of longitudinal follow-up. Table 2 provides additional details on the surveys assessed in the mobile protocol. After each survey, participants complete 3 brief performance-based cognitive tasks (described below; screenshots provided in Figures2  3, and Figure 4). At the end of the 14 days of daily diary, participants complete an “End of Diary” survey in which they complete an additional brief set of questionnaires and rate their satisfaction and ease of use when participating in the mobile protocol. Upon completion of the protocol, participants return the mobile device and charger to our university using a self-addressed mailer box or in-person delivery at a community center.

**Table 2.** Mobile protocol measures.

Target and construct	Response option	Items and operational detail	Relevant reference
Contextual factors			
Location	Radio button	Where are you right now? (My home, other person’s home, community center, etc.)	55
Social company	Multiple options	Who are you with right now? (No one, spouse or partner, friend(s), pet(s), etc)	55
Daily Inventory of Stressful Events (DISE)			
Stressor exposure	Yes or no	Composite across 6 stressor types	54
Stressor type	Yes or no	Arguments, avoided arguments, work, home, discrimination, network, and other	56
Who was involved	Multiple options	Spouse/partner, child, parent, sibling, friend, coworker, etc	57
When did it happen	Yesterday or today; AM or PM	Yesterday versus today; what time of day?	58
Stressor appraisals			
Severity	None at all-very	How stressful was this for you?	59
Control	None at all-a lot	How much control did you have over the situation?	60
Resolution	Yes or no	Is the situation resolved?	61
Resource risk	None at all-a lot	Financial, health and safety, schedule, and socioemotional	62
Stressor reactivity	Calculated slope	Emotional, behavioral, or biological reactions to stressors on the same day	63
Stressor residue	Calculated slope	Prolonged responses extending to the following day	61
Stressor diversity	Calculated index	Dispersion of stressors across multiple domains; can it be calculated using Shannon’s entropy index?	64
Anticipatory stress	Not at all-very	How stressful do you expect tomorrow to be?	65
Daily events			
Positive events	Yes or no	Interaction, work, home, network, and other	66
Who was involved	Multiple options	Spouse or partner, child, parent, sibling, friend, coworker, etc	66
When did it happen	AM or PM	What time of day?	66
Anticipatory pleasantness	Not at all-very	How pleasant do you expect tomorrow to be?	66
Support exchanges	Yes or no	Give and/or receive unpaid assistance and emotional support	67
Received care	Multiple options	Did you need help in any of the following areas? (physical, financial, etc)	48
Who was involved	Multiple options	Paid caregiver or unpaid caregiver (family, friend, etc)	48
Time providing care	Hours	How many hours did they spend helping you?	48
Provided care	Multiple options	Did you provide help in any of the following areas? (physical, financial, etc)	48
Who was involved	Multiple options	As a paid caregiver or an unpaid caregiver (family, friend, etc)	48
Time providing care	Hours	How many hours did you spend helping them?	48
Everyday discrimination	Yes or no	Nine items with follow-up reasons for discrimination (eg, race, gender, and age)	68
Time use			
Work, volunteer, and unpaid assistance	Hours and minutes	How much time is spent working, volunteering, and providing unpaid assistance?	69
Physical activity, leisure, TV, and sitting	Hours and minutes	How much time is spent on physical activity, leisure, TV, and sitting?	70
Daily psychosocial experiences			
Negative affect	None of the time-all of the time	Fourteen items for “How much of the time today did you feel (affect item)?”	71
Positive affect	None of the time-all of the time	Thirteen items for “How much of the time today did you feel (affect item)?”	71
Quiet ego	Not at all-a great deal	Four items on quiet ego (eg, “Today, I felt aware and less judgment of myself as well as others.”)	72
Daily physical health indices			
Health behaviors
Cigarette smoking	Quantity	How many cigarettes did you smoke?	73
Vape and e-cigarettes	Hours and minutes	How much time is spent vaping or using e-cigarettes?	74
Alcohol drinks	Quantity	How many drinks did you have?	75
Fast food	Yes and no	Did you eat at a fast-food restaurant?	76
Self-rated physical symptoms
Experience	Yes and no	Headache, fatigue, fever, muscle weakness, chest pain, etc	
Severity	Very mild-very severe	How severe was this symptom?	58
Sleep quantity and quality	Hours and minutes	What time did you wake up today? How many hours of sleep did you get last night?	77
Sleep quality	Very poor-very good	How would you rate the quality of your sleep last night?	78
Daily cognitive health			
Memory lapses
Frequency	Yes and no	Forget errand, medication, appointment, and someone’s name	79
Irritation	Not at all-very much	How much did forgetting this bother you?	79
Interference	Not at all-very much	How much did forgetting this interfere with your routine?	79
Unconstructive repetitive thoughts	None of the time-all of the time	Think about personal problems, trouble concentrating, etc	80
Mobile cognitive tasks
Symbol match	Response time	~60-second task measuring processing speed	65
Grid memory	Error score	~60-second task measuring spatial working memory	65
Color shapes	Hit and error percentage	~60-second task measuring memory binding	55

**Figure 3.**
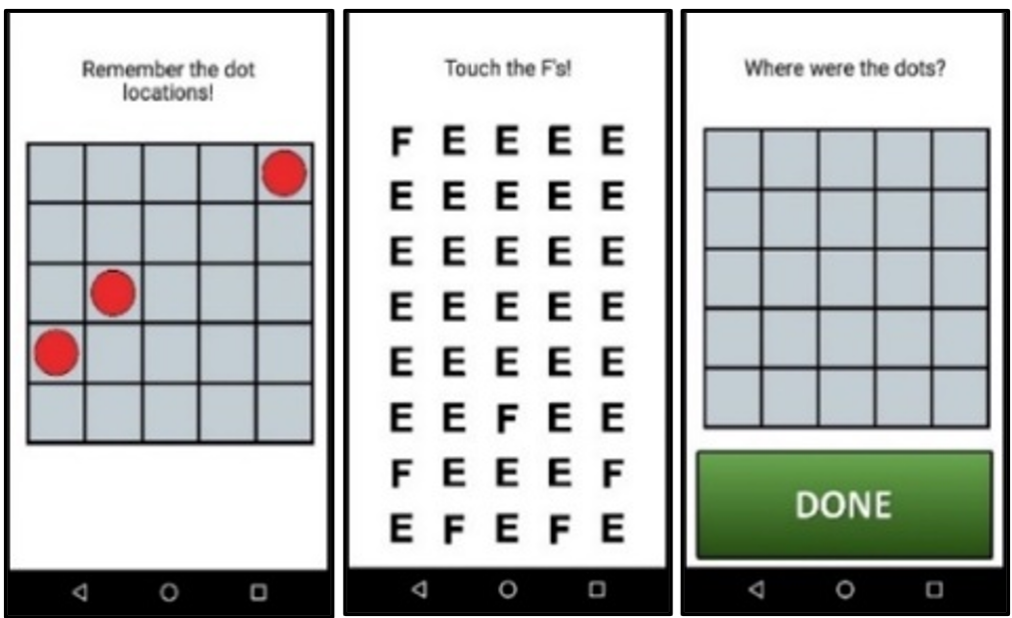
Screenshots of the grid memory mobile cognitive task. Participants complete this cognitive task during the evening assessment for 14 consecutive days.

**Figure 4.**
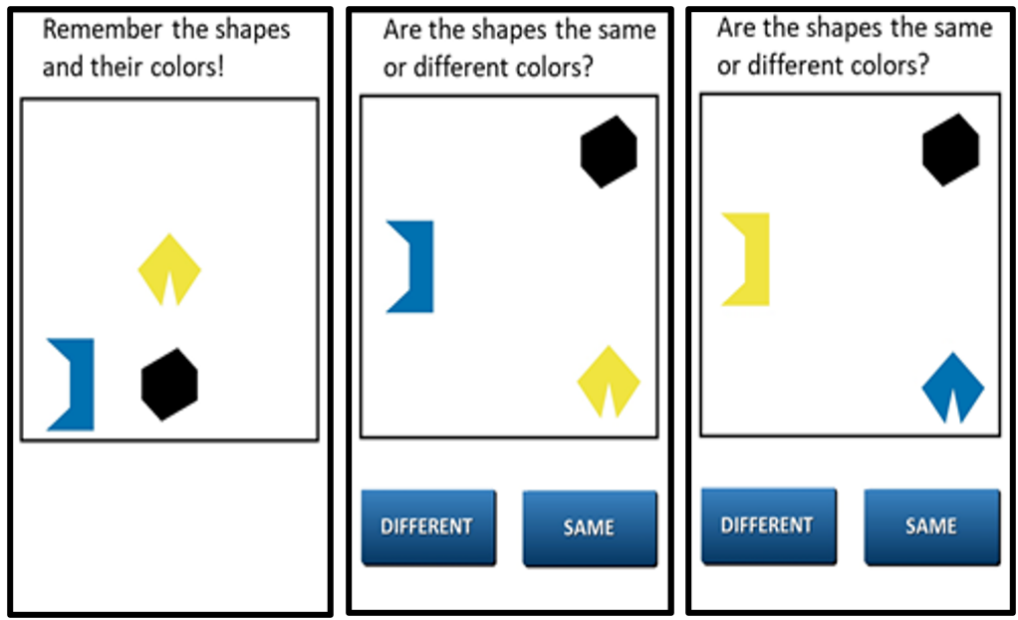
Screenshots of the color shapes mobile cognitive task. Participants complete this cognitive task during the evening assessment for 14 consecutive days.

The mobile device includes 3 tasks assessing processing speed, spatial working memory, and memory binding. These tasks were selected based on their reliability when administered repeatedly over a brief time interval [81] and their sensitivity to both daily contextual factors [65] and AD [82]. Completion of these tasks takes 3‐4 minutes following the survey. Adherence in completing these tasks during a previous study among diverse older adults with MCI and those who were cognitively unimpaired was over 80% [55].

The processing speed task requires participants to compare 2 symbol pairs at the top of the screen with 2 symbol pairs at the bottom of the screen and decide as quickly as possible which of the bottom-screen pairs matches a top-screen pair. In the spatial working memory task, participants memorize the location of 3 red dots that appear on a 5×5 grid for 3 seconds. After an 8-second visual distractor, participants then recall their locations. The memory binding task requires participants to memorize the shapes and colors of 3 different colored polygons for 3 seconds. Participants then must recognize whether newly presented polygons are identical to the originals. Collectively, performance on these tasks represents the 3 dependent variables of cognitive health examined in Aim 2.

Our naturalistic assessment of performance-based mobile cognitive health situates the NAZMS protocol within the National Institutes of Health (NIH)-funded Mobile Monitoring of Cognitive Change (M2C2) assessment infrastructure. M2C2 is an open-source and flexible assessment infrastructure designed to advance accurate and sensitive measurement of cognitive change via mobile assessments (for more information, refer to [83]). The M2C2 mobile tests are being used in a wide range of NIH-funded studies, including the Einstein Aging Study, which is using these mobile assessments in a longitudinal study of MCI and dementia risk (eg, [55]), as well as the newest wave of data collection currently underway for the National Study of Daily Experiences, which is using these mobile assessments to monitor psychosocial and developmental contributors to cognitive health across a national adult lifespan sample. This assessment infrastructure facilitates cross-study harmonization of cognitive testing and direct comparison of M2C2 cognitive tests across NAZM’s rural sample of Northern Arizonans and different NIH-funded aging studies across the United States.

### Planned Analyses

Data from qualitative interviews will provide dyad preferences for identifying and monitoring MCI and SCD symptoms and for engaging in remotely delivered supportive interventions to contend with or reduce symptoms and associated problems of cognitive impairment. These interviews will also reveal information on essential components of a culturally appropriate intervention for rural ADRD dyads for intervention development, including the types of technology and comfort level with technologies for the detection of cognitive impairment and receiving support. The interviews may further inform the roles of technology access (eg, internet and broadband availability), comfort level with using technologies, and preferences toward written materials, digital tools, or online supports across different rural communities in Northern Arizona. For example, we may learn preferences that help inform a future pilot intervention for brain health support that could include combinations of written and digital-based screening, interactive features via website or mobile apps, accessible training materials, as well as live support or coaching. This is an important consideration for rural communities in the mountainous region of Northern Arizona, where the signal may be comparatively weaker or less consistent than the signal in more densely populated cities. Data collection will continue until saturation [32,84]. Data analysis will be informed by the principles of interpretative description [85], which include an emphasis on practical application of findings for practice. Interviews are audio recorded, professionally transcribed, quality-checked for accuracy, and analyzed using Dedoose (version 10.0.25; SocioCultural Research Consultants, LLC [86]). Development of analytic codes will be based on the framework method, which is recommended for qualitative health research and can be adapted for both deductive and inductive coding [87]. We plan to use deductive coding based on established models of technology acceptance [30,33] and dyadic processes [31], as well as inductive coding to understand unique and emergent patterns in the data provided by our analytic sample of rural dyads in Northern Arizona. Study interviewers will maintain field notes to provide context for data analysis. We will develop and adhere to a protocol to promote the credibility, transferability, dependability, and confirmability of qualitative findings [88].

Data from the quantitative protocol will specify for whom (ie, between-person) and on which days (ie, within-person) modifiable factors are related to better daily cognitive health. Our 14-day digital health approach repeatedly measures cognitive health in people’s natural environments, improving ecological validity, reducing recall bias, and enabling examination of unique signatures of variability in cognitive performance not possible in conventional single-shot assessments completed in clinics [55]. We will use multilevel models (MLMs) with maximum likelihood estimation to accommodate the nested structure of the data (14 days nested within people) and intermittent missing data using SAS (version 9.4; SAS Institute [89]). Day-level within-person deviation scores for psychosocial, sociocultural, and contextual social determinants will be computed by subtracting an individual’s average amount of the variable from their daily score [90,91]. Within-person deviation scores facilitate examination of which days modifiable protective and risk factors are related to better (or worse) cognitive health. Models will be adjusted for systematic trends across days, as well as relevant covariates (eg, caregiver strain and MoCA Blind score) and adherence rate to the protocol (to adjust for potential missing data patterns). Separate MLMs will be estimated for each cognitive outcome. To examine relationships between caregiver factors, dyadic factors, and the care receiver’s cognitive health, we will regress each cognitive health outcome on the caregiver factors and dyadic factors assessed at baseline. To examine daily influences of modifiable protective and risk factors on cognitive health among individuals with MCI, the day-level cognitive health outcomes will be regressed on the day-level within-person deviation scores for psychosocial, sociocultural, and contextual factors.

### Sample Size Considerations

Our initial target sample size is 30 rural care dyads. We determined 30 rural care dyads as a starting point for NAZMS data collection based on its mixed methods design components (qualitative and quantitative), the dyadic nature of interviews (30 caregivers and 30 participants with cognitive impairment), and statistical power analysis for quantitative analyses. For the qualitative arm, 30 dyads will be sufficient to adequately address our research questions and reach saturation based on our theoretical grounding and qualitative analytic framework [92,93]. For the quantitative arm, statistical power for quantitative protocol analyses among the target 30 participants with cognitive impairment was determined using techniques recommended by Cohen [94] for determining power to detect effects using repeated-measures ANOVA and NAZMS details. Our proposed MLM approach is more powerful than repeated-measures ANOVA and uses all available data from up to 14 measurements; therefore, this calculation provides a conservative estimate of power to detect effects. Power analysis was conducted using G*Power (Franz Faul, Edgar Erdfelder, Albert-Georg Lang, and Axel Buchner [95]), power=0.80, target (n=30), and *α*=.05. We have 80% power to detect small-to-medium effects of daily predictors for cognitive health outcomes (Cohen *f*=0.15).

Our team intends to expand NAZMS data collection capacity in subsequent years as we learn from this initial target sample. Specifically, our goal is to expand the NAZMS sample size beyond these initial 30 dyads to build our capacity to better characterize supports for people with cognitive impairment and their family members in rural communities across the Southwest.

### Ethical Considerations

All participants provided written or verbal (if administered remotely via Zoom) informed consent and received compensation for their participation. All procedures were approved by our university’s Institutional Review Board (IRB; approval no. 2090130). Participants were eligible to receive up to US $100 in gift cards for their participation (US $50 after completion of the qualitative interviews and surveys and an additional US $50 upon completion of the daily diary protocol).

## Results

As of September 2025, 39 adults have participated in cognitive screenings. Of the 39 adults, 17 have screened positive for MCI, and 38 have screened positive for SCD. Eleven dyads have been recruited to complete the NAZMS protocol with plans to continue recruitment this year and expand data collection capacity in subsequent years. Results from this study are expected to be published by summer 2027.

## Discussion

### Implications for Rural Communities

Through the project activities and subsequent data analysis, there are multiple implications for care dyads at risk for ADRD living in memory care deserts and rural areas. First, we will determine culturally appropriate methods and technologies for identifying rural families facing cognitive impairment in community-based settings (Aim 1). Second, through the convergence of interviews and daily cognitive assessment over 14 days, we will be able to characterize rural caregiver-receiver dyads with information from not only intervention content and engagement preferences, but also the modifiable protective and risk factors that influence cognitive health in everyday life (Aim 2). Third, we will establish the content needed to develop an intervention for this population (Aim 1). Fourth, we will establish collaborative relationships with regional community centers and Tribal Nations that will be needed for future research and resource distribution with rural ADRD care dyads (Aim 1). Finally, we will share what we learn through community forum events and brain health workshops designed to educate rural communities by confronting stigma and anxiety associated with cognitive impairment and to promote cognitive screenings and the importance of early detection (Aims 1 and 2).

### Implications for Designing Digital Health Technologies

Digital health tools and interventions can be viable ways to support people with cognitive impairment [96] and caregivers [97]. However, the needs and preferences for different digital health technologies vary depending on cognitive status and caregiving demands [98], with recent systematic reviews and meta-analyses calling for future research to address issues around accessibility, acceptability, and sustainability of digital health interventions in these populations [97] and rural communities [99]. For example, a mixed methods evaluation of a web-based platform designed to improve the quality of life for people with cognitive impairment and reduce burden for caregivers recognized the need for end users to be involved in the development of the digital health tools [98]. Our dyadic data collection, intentionally connecting with both members of the care dyad, will address the gaps outlined by this previous research and situate NAZMS in a broader literature of digital health technologies that can support both people with cognitive impairment and their family members living in rural communities.

### Developing Partnerships With Tribal Nations for Future Collaboration

We are in the process of developing partnerships with Tribal Nations to provide the NAZMS protocol to Indigenous communities residing on Tribal lands. In spring 2025, we received conditional approvals from our university’s Office of Native American and Indigenous Advancement’s Tribal Consultation process and IRB to seek formal Tribal IRB approval that can expand recruitment to Tribal lands in the future. Building on culturally informed frameworks [100] and community-engaged approaches with Tribal Nations [101], we look forward to examining unique protective and risk factors for ADRD in Indigenous communities [102] and supporting Indigenous care dyads in the Southwest.

### Conclusions

The NAZMS dyadic mixed methods digital health protocol aims to support rural families at risk for ADRD by understanding intervention preferences and identifying the modifiable protective and risk factors that influence cognitive health in everyday life. The findings will respond to national priorities in ADRD research [27] for the development of community-based education programs and the use of digital assessments of cognitive health and well-being [1].

## Abbreviations

AD: Alzheimer disease
ADRD: Alzheimer disease and related dementias
IRB: Institutional Review Board
M2C2: Mobile Monitoring of Cognitive Change
MCI: mild cognitive impairment
MLM: multilevel model
MoCA: Montreal Cognitive Assessment
NAZMS: Northern Arizona Memory Study
NIH: National Institutes of Health
SCD: subjective cognitive decline
